# Association between plantar flexor muscle volume and dorsiflexion flexibility in healthy young males: ultrasonography and magnetic resonance imaging studies

**DOI:** 10.1186/s13102-021-00233-z

**Published:** 2021-01-29

**Authors:** Tadashi Suga, Masafumi Terada, Keigo Tomoo, Yuto Miyake, Takahiro Tanaka, Hiromasa Ueno, Akinori Nagano, Tadao Isaka

**Affiliations:** 1 Faculty of Sport and Health Science, Ritsumeikan University, 1-1-1 Nojihigashi, 525-8577 Kusatsu, Shiga Japan; 2 Graduate School of Health and Sport Science, Nippon Sport Science University, Fukasawa, Setagaya-ku, Tokyo Japan; 3 Research Fellow of Japan Society for the Promotion of Science, Kojimachi, Chiyoda-ku, Tokyo Japan

**Keywords:** Range of motion, Stiffness, Ultrasonography, Magnetic resonance imaging

## Abstract

**Background:**

Although joint flexibility is important for human locomotion, the determinants of joint flexibility are not fully understood. In this study, we examined the relationship between dorsiflexion flexibility and plantar flexor muscle size in healthy young males.

**Methods and results:**

The dorsiflexion flexibility was assessed using range of motion (ROM) and stiffness during active and passive dorsiflexion. Active ROM was defined as the maximal angle during voluntary dorsiflexion. Passive ROM was defined as the angle at the onset of pain during passive dorsiflexion. Passive stiffness was calculated as the slope of the linear portion of the torque-angle curve between 10º and 20º dorsiflexion of the ankle during passive dorsiflexion. In the first study, the plantar flexor muscle volume (MV) in 92 subjects was estimated on the basis of the lower leg length and plantar flexor muscle thickness, as measured using ultrasonography. The estimated plantar flexor MV correlated significantly with active ROM (*r* = -0.433), passive ROM (*r* = -0.299), and passive stiffness (*r* = 0.541) during dorsiflexion (*P* = 0.01 for all). In the second study, the plantar flexor MV in 38 subjects was measured using magnetic resonance imaging. The plantar flexor MV correlated significantly with plantar flexor active ROM (*r* = -0.484), passive ROM (*r* = -0.383), and passive stiffness (*r* = 0.592) during dorsiflexion (*P* = 0.05 for all).

**Conclusions:**

These findings suggest that a larger plantar flexor MV is related to less dorsiflexion flexibility in healthy young males.

## Background

Joint flexibility is an important factor for performing physical activity and maintaining good health in adults of all ages [1]. Additionally, we and others previously determined that joint flexibility is related to athletic performance in athletes, including endurance runners and sprinters [2–5]. Therefore, the identification of the factors determining joint flexibility may be useful in understanding physical function and athletic performance in various populations.

Range of motion (ROM) is a universal parameter used for assessing joint flexibility [6]. Additionally, passive stiffness, usually calculated as the slope of the linear portion of the torque-angle curve during passive joint movement, has been used as a joint flexibility parameter [4, 5, 7–12]. Although the determinant(s) of these joint flexibility parameters are still not fully understood, they have been shown to be affected by some factors, including morphological factors [7, 9, 10, 12].

Several studies have reported that there is a relationship between joint flexibility and agonist muscle size in several joints [7, 9, 10, 12]. In the upper limb, using ultrasonography (US), Chleboun et al. [7] determined that a larger elbow flexor muscle volume (MV) is correlated with higher passive elbow extension stiffness. In the lower limb, Kubo et al. [9] reported that a larger muscle thickness (MT) at the posterior portion of the lower leg (i.e., plantar flexors), as measured using US, is correlated with higher passive dorsiflexion stiffness. Moreover, using US, Ryan et al. [12] reported the same result by showing the relationship between larger US-measured plantar flexor cross-sectional area (CSA) and higher passive dorsiflexion stiffness. These findings suggest that a larger muscle size may be associated with limited flexibility in both the upper and lower limb joints. Nevertheless, magnetic resonance imaging (MRI) is known to be a more appropriate tool for measuring muscle size than US [13–16]. Additionally, muscle volume (MV) is a more appropriate marker for evaluating muscle size than MT and CSA [13–15]. Only one study using MRI, by Magnusson et al. [10], reported that a larger knee flexor CSA is correlated with higher passive knee extension stiffness; however, the authors did not measure MV. Therefore, to determine the relationship between muscle size and passive stiffness, it is necessary to measure MV using MRI.

Comparted to the amount of information available on the relationship between muscle size and passive stiffness, little is known about whether muscle size is related to ROM. To the best of our knowledge, only one study, by Magnusson et al. [10], determined that there is a correlation between larger knee flexor CSA and lower passive knee extension ROM. In addition, although ROM is largely divided into active and passive ROMs, no study has examined the relationship between muscle size and active ROM. Overall, the relationships between muscle size and joint flexibility parameters, especially active and passive ROMs, are not fully understood. To clarify this issue, in the present study, we aimed to determine the relationships between MV, which was estimated or measured using US and MRI, and three joint flexibility parameters (i.e., active ROM, passive ROM, and passive stiffness). We first examined the relationships between US-estimated plantar flexor MV and dorsiflexion flexibility in a relatively large study population (i.e., study 1). Thereafter, to verify this relationship, we examined the relationship between plantar flexor MV and dorsiflexion flexibility using MRI (i.e., study 2), since MRI yields a higher quality measurements for MV evaluating MV than does US [13–15].

## Methods

### Subjects

Ninety-two healthy, young males (age: 21.5 ± 2.3 years) participated in study 1 of this study. Thirty-eight (age: 21.6 ± 1.4 years) healthy, young males also participated in study 2 of this study. The subjects of study 2 were those who participated in study 1 and obtained consent to participate in study 2. The subjects were recreationally active but were not involved in any specific physical training programs, which include flexibility training, within the previous 3 years. Nevertheless, many of them had participated in recreational sports and/or physical training for 2–3 hours per week. The subjects who participated in studies 1 and/or 2 had no history of major injuries and previous surgery of the legs, and were free from any known orthopedic, neurological, and neuromuscular problems. The subjects who participated in study 2 had no contraindications to MRI. Subjects that did not meet these inclusion criteria were excluded from this experiment. All subjects were informed of the experimental procedures and provided written consent to participate in the study. All procedures were approved by the Ethics Committee of Ritsumeikan University (BKC-IRB-2016-047).

### Joint flexibility parameters

**Fig. 1:**
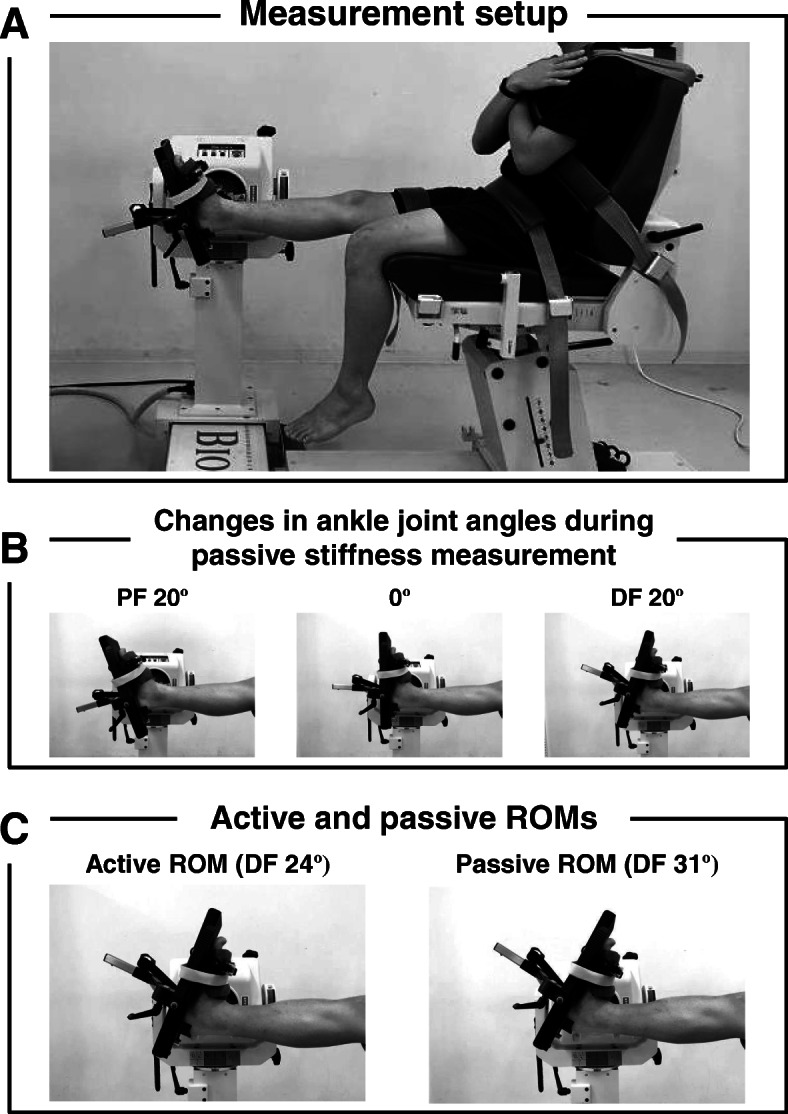
**Experimental photographic images for measuring dorsiflexion flexibility parameters** Three dorsiflexion (DF) flexibility parameters of the right leg were measured in a sitting position with full extension of the knee joint. The foot was tightly strapped to a footplate connected to the lever arm of a dynamometer. Panel **a** is a photographic image of the setup for measuring three DF flexibility parameters. Panel **b** contains photographic images of the changes in ankle joint angles, including plantar flexion (PF) 20º, 0º, and DF 20º, during the passive stiffness measurement. Panel **c** contains photographic images of the maximal angles during the active and passive range of motion (ROM) measurements. The angles of active and passive DF ROMs were at DF 24º and 31º, respectively

Experimental photographic images for measuring three dorsiflexion flexibility parameters are presented in Fig. 1. The three dorsiflexion flexibility parameters of the right leg were measured using a dynamometer system (BIODEX system 3; BIODEX Medical, Shirley, NY, USA). The dynamometer system has been frequency used to measure joint flexibility parameters, especially passive ROM and passive stiffness [4, 5, 8–12, 17–22]. Prior to the measurement of dorsiflexion flexibility, subjects were instructed to refrain from stretching exercise for at least 2 hours. Previous studies have reported that changes in dorsiflexion flexibility parameters (e.g., passive ROM and stiffness) after acute plantar flexor stretching exercises reversed to that before stretching exercises within 30 min [11, 19, 22]. Thus, we considered that this interval to refrain from stretching exercises in the present study was sufficient to measure joint flexibility. In addition, the subjects were instructed to avoid strenuous physical activity within 24 hours before the dorsiflexion flexibility measurements. Furthermore, to minimize the effects of difference in physical activity in the experimental day on dorsiflexion flexibility among subjects, the measurements of dorsiflexion flexibility parameters in all subjects were performed at approximately the same time (i.e., between 09:00 and 11:00 am) in the morning.

Subjects were placed in a sitting position with full extension at the knee joint on a dynamometer. The hip was securely fixed by seat belts. The foot was tightly strapped to a footplate connected to the lever arm of a dynamometer. To measure active ROM, ankle joint of the subjects was set at a 20º plantar flexion of the ankle joint, and then subjects were requested to performing voluntary dorsiflexion as slowly as possible. Angles during active dorsiflexion were continuously sampled at 100 Hz via a dynamometer. The active ROM was measured three times. A highest maximal dorsiflexion angle among the three measurements was defined as active dorsiflexion ROM.

To measure passive ROM and stiffness, subjects were requested to stay as relaxed as possible and not provide any voluntary resistance. Passive dorsiflexion of the ankle joint was performed from 20º plantar flexion to maximal dorsiflexion angle. The maxima dorsiflexion angle was assessed as the onset of pain when the musculotendinous tissues were stretched. A constant angular velocity during passive dorsiflexion was set to 2º/s, which was the lowest angular velocity of the dynamometer system used in this study, to inhibit the stretch reflex, as in our and other previous studies [4, 5, 17–21]. During the passive dorsiflexion, torques were continuously sampled at 100 Hz via a dynamometer. A maximal angle during passive dorsiflexion was defined as passive dorsiflexion ROM. Passive dorsiflexion stiffness was calculated as the linear slope of the torque-angle curve between 10º and 20º dorsiflexion of ankle joint, as in our previous studies [4, 5].

### US-estimated plantar flexor MV (study 1)

The lower leg length of the right leg was measured as the distance between the lateral malleolus of the fibula and the lateral condyle of the tibia. The lower leg circumference was measured at the proximal 30 % of the lower leg length. The plantar flexor MT was measured at a similar position to the lower leg circumference using US (IPC-1530; Aloka, Tokyo, Japan), as in our and other previous studies [15, 23]. The plantar flexor MV was estimated on the basis of the lower leg length and plantar flexor MT using the following equation: MV = 218.1 ⋅ MT (cm) + 30.7 ⋅ lower leg length (cm) − 1730.4). This equation was developed with variation and cross-variation studies by Miyatani et al. [15].

### MRI-measured plantar flexor MV (study 2)

The MRI measurements of the right lower leg were performed using a 1.5-T magnetic resonance system (Signa HDxt; GE Medical Systems, WI, USA). Subjects were placed in a supine position on the scanner bed, with both knees fully extended and both ankles set at the neutral position (i.e., 0°). To prevent the deformation of the plantar flexor muscles, it was measured in a condition in which the ankle was held up, and the calf was allowed to be free from the bed.

Axial T_1−_weighted MRI scans of the lower leg were acquired with a standard body coil. The axial scans were obtained in successive slices with an inter distance of 10 mm with a repetition time of 600 ms, echo time of 7.6 ms, field of view of 480 mm, and matrix size of 512 × 256 pixels. From these scans, CSAs on each slice in the plantar flexor muscles were measured using image analysis software (OsiriX Version 5.6, Switzerland). The plantar flexor muscles included the gastrocnemius lateralis, gastrocnemius medialis, and soleus. The adipose and connective tissue incursions were excluded as much as possible from each image of the plantar flexor CSAs. The maximum CSAs were adopted as the plantar flexor anatomical CSA (ACSA). The plantar flexor MV was calculated by multiplying the sum of the CSAs along their length at intervals of 10 mm.

### Statistical analysis

All data are expressed as mean ± SD. In both studies 1 and 2, relationships between measured variables were assessed using a Pearson’s product moment correlation coefficient. Statistical significance level was defined at *P* < 0.05. All statistical analyses were conducted using IBM SPSS software (version 19.0; International Business Machines Corp, NY, USA).

## Results

### Relationships between US-estimated plantar flexor MV and dorsiflexion flexibilities

Mean values in measured variables obtained in study 1 are listed in Table 1. Active ROM correlated significantly with passive stiffness. (*r* = -0.637, *P* < 0.001). Similarly, passive ROM correlated significantly with passive stiffness (*r* = -0.436, *P* < 0.001).

**Table 1:** Mean values of measured variables obtained in study 1 (n = 92)

	Mean ± SD	Range
Body height, cm	171.6 ± 5.6	158.0−186.5
Body mass, kg	65.4 ± 7.7	52.0−89.7
Body mass index, kg/m^2^	22.2 ± 2.5	17.8−30.3
Active ROM, °	23.8 ± 5.9	6.0−35.0
Passive ROM, º	30.5 ± 6.6	20.0−47.0
Passive stiffness, Nm/°	0.80 ± 0.35	0.30−2.01
Lower leg length, cm	39.8 ± 2.1	35.5−46.5
Lower leg circumference, cm	36.6 ± 2.0	30.8−42.5
Plantar flexor MT, cm	6.9 ± 0.5	5.7−8.2
Estimated plantar flexor MV, cm^3^	987.1 ± 129.7	670.5−1286.4

*ROM* range of motion; *MT* muscle thickness, *MV* muscle volume

Correlation coefficients between plantar flexor morphological variables and dorsiflexion flexibility parameters obtained in study 1 are summarized in Table 2. The lower leg length did not correlate with all three dorsiflexion flexibility parameters. By contrast, the lower leg circumference and plantar flexor MT correlated significantly with the three dorsiflexion flexibility parameters (*r* = -0.447 and − 0.415, respectively, for active ROM; *r* = -0.484 and − 0.254, respectively, for passive ROM; *r* = 0.523 and 0.510, respectively, for passive stiffness; *P* < 0.05 for all). Similarly, the estimated plantar flexor MV correlated significantly with three flexibility parameters (*r* = -0.443 for active ROM, *r* = -0.299 for passive ROM, *r* = 0.541 for passive stiffness; *P* < 0.01 for all).

**Table 2:** Correlation coefficients between morphological variables and flexibility parameters obtained in study 1

	Active ROM		Passive ROM		Passive stiffness	
	*r*	*P* value	*r*	*P* value	*r*	*P* value
Lower leg length	−0.134	0.203	−0.152	0.149	0.183	0.080
Lower leg circumference	**−0.447**	**<0.001**	**−0.484**	**<0.001**	**0.523**	**<0.001**
Plantar flexor MT	**−0.415**	**<0.001**	**−0.254**	**0.014**	**0.510**	**<0.001**
Estimated plantar flexor MV	**−0.433**	**<0.001**	**−0.299**	**0.004**	**0.541**	**<0.001**

Bold fonts indicate significant correlations (*P* < 0.05) between flexibility parameters and morphological variables

### Relationships between MRI-measured plantar flexor MV and dorsiflexion flexibilities

Mean values in measured variables obtained in study 2 are listed in Table 3. Active ROM correlated significantly with passive stiffness. (*r* = -0.486, *P* = 0.002). Similarly, passive ROM correlated significantly with passive stiffness (*r* = -0.404, *P* = 0.012).

**Table 3:** Mean values of measured variables obtained in study 2 (n = 38)

	Mean ± SD	Range
Body height, cm	171.4 ± 5.2	161.2−186.0
Body mass, kg	65.5 ± 8.5	52.0−89.7
Body mass index, kg/m^2^	22.3 ± 2.7	17.8−30.3
Active ROM, °	24.9 ± 5.7	11.0−35.0
Passive ROM, º	30.1 ± 7.3	20.0−47.0
Passive stiffness, Nm/°	0.71 ± 0.33	0.30−1.75
Lower leg length, cm	39.4 ± 2.1	36.5−46.5
Lower leg circumference, cm	36.7 ± 2.2	30.8−42.5
Plantar flexor ACSA, cm^2^	46.1 ± 6.6	29.9−63.5
Plantar flexor MV, cm^3^	755.2 ± 115.8	511.0−1108.5

*ACSM* anatomical cross-sectional area

Correlation coefficients between plantar flexor morphological variables and dorsiflexion flexibility parameters in study 2 are summarized in Table 4. The lower leg length did not correlate with all three dorsiflexion flexibility parameters. By contrast, the lower leg circumference and plantar flexor ACSA correlated significantly with the three dorsiflexion flexibility parameters (*r* = -0.552 and − 0.438, respectively, for active ROM; *r* = -0.638 and − 0.382, respectively, for passive ROM; *r* = 0.610 and 0.598, respectively, for passive stiffness; *P* < 0.05 for all). Similarly, the plantar flexor MV correlated significantly with three flexibility parameters (*r* = -0.484 for active ROM, *r* = -0.383 for passive ROM, *r* = 0.592 for passive stiffness; *P* < 0.05 for all).

**Table 4:** Correlation coefficients between morphological variables and flexibility parameters obtained in study 2

	Active ROM		Passive ROM		Passive stiffness	
	*r*	*P* value	*r*	*P* value	*r*	*P* value
Lower leg length	0.022	0.896	−0.108	0.519	0.114	0.495
Lower leg circumference	**−0.522**	**0.001**	**−0.638**	**<0.001**	**0.610**	**<0.001**
Plantar flexor ACSA	**−0.438**	**0.006**	**−0.382**	**0.018**	**0.598**	**<0.001**
Plantar flexor MV	**−0.484**	**0.002**	**−0.383**	**0.018**	**0.592**	**<0.001**

Bold fonts indicate significant correlations (*P* < 0.05) between morphological variables and flexibility parameters

## Discussion

In this study, we first determined that a larger US-estimated plantar flexor MV is correlated with less dorsiflexion flexibility, as evaluated using three parameters. Thereafter, to verify this finding, we examined the correlations between plantar flexor MV and dorsiflexion flexibility parameters using MRI and demonstrated that a larger MRI-measured plantar flexor MV is correlated with less dorsiflexion flexibility. Several previous studies have reported that a larger muscle size, as measured using US or MRI, is related to less joint flexibility (i.e., lower ROM and higher passive stiffness) [7, 10]. However, prior to the present study, no study has examined the relationship between MRI-measured MV and joint flexibility. Therefore, the present study is the first to determine this relationship.

Most previous studies examining the relationship between muscle size and joint flexibility employed passive stiffness as a parameter of joint flexibility [7, 9, 10, 12]. Only one study, by Magnusson et al. [10], determined that a larger biceps femoris CSA is correlated with lower passive knee extension ROM. The present study corroborates their results by showing the relationship between larger plantar flexor MV and lower passive dorsiflexion ROM. Furthermore, in addition to passive ROM, the present study assessed active ROM and demonstrated the relationship between larger plantar flexor MV and lower active dorsiflexion ROM. The magnitude of the passive ROM may affect pain sensitivity (i.e., stretch tolerance) when musculotendinous tissues are stretched among subjects [20, 21]. In contrast, the magnitude of active ROM does not appear to have this effect. Therefore, active ROM in addition to passive ROM and passive stiffness is a useful parameter to assess joint flexibility.

This study determined that the plantar flexor MV is inversely related to three dorsiflexion flexibility parameters. Nevertheless, the strength of these relationships was moderate; thus, the plantar flexor MV alone can explain only a part of dorsiflexion flexibility. Recently, shear wave ultrasound elastography has been used to assess the stiffness of tissues, including muscle [17, 19–21, 24]. Previous studies have determined that higher plantar flexor muscle stiffness measured using shear wave ultrasound elastography is correlated with lower dorsiflexion ROM in healthy young adults [20, 21]. To further clarify the present findings, further studies are needed to comprehensively determine the effects of the morphological and mechanical properties of muscle tissue on joint flexibility.

In the findings of study 1, correlation coefficients with all three dorsiflexion flexibility parameters were relatively higher for US-estimated plantar flexor MV than for plantar flexor MT. The findings of study 2 also showed that although correlation coefficients with passive dorsiflexion ROM and stiffness were similar between plantar flexor MV and ACSA, the correlation coefficient with active dorsiflexion ROM was relatively higher for plantar flexor MV than for plantar flexor ACSA. These present findings suggest that MV may be a better marker to evaluate the relationship between muscle size and joint flexibility than MT and CSA (i.e., ACSA). Nevertheless, in both studies 1 and 2, we found that the correlation coefficients with all three dorsiflexion flexibility parameters were relatively higher for lower leg circumference than for plantar flexor MV. The lower leg circumference includes not only muscle tissue properties but also properties of other tissues, including the fascia, adipose, and skin tissues. Of those tissues, Yoshitake et al. [24] determined that the skin may affect shear wave ultrasound elastography-measured muscle stiffness; the authors showed that the muscle stiffness; decreased by 50 % after the skin covering the gastrocnemius medialis was removed in cadavers. In the clinical setting, MV is often inconvenient to measure, especially using MRI, owing to the large clinical demand and considerable costs involved. Therefore, the findings of the present and previous studies suggest that the lower leg circumference may be useful as a sufficient and surrogate marker to assess joint flexibility in various large populations.

This study recruited only healthy young males, and therefore, it remains unclear whether the present findings can be generalized to populations of other ages (i.e., older individuals) and with different health states (i.e., patients with chronic diseases) or females. In particular, older individuals have less joint flexibility than do young individuals [8, 25], despite reductions of muscle size, because of age-related physiological processes [26]. Thus, factors other than muscle size may play an important role in determining joint flexibility in older individuals. Indeed, previous studies have demonstrated that the sciatic nerve may potentially limit dorsiflexion ROM [17, 20]. In a recent study, Hirata et al. [20] found that stiffness of the sciatic nerve (i.e., measured at 15º dorsiflexion) measured using shear wave ultrasound elastography is correlated with passive dorsiflexion ROM in older individuals but not young individuals. This finding may be due to changes in the sensitivity to tension applied to the sciatic nerve that occur with aging because sciatic nerve stiffness is lower in older individuals than in young individuals [20]. In addition, we and others previously determined that higher passive dorsiflexion stiffness is correlated with better race performance (i.e., 100-m or 5000-m personal best times) in both sprinters and endurance runners [2–5]; thus, this result may be simply due to the relationship between muscle size and race performance in both athlete groups. Although several studies have reported a potential relationship between plantar flexor muscle size and sprint performance [27, 28], we and others determined the lack of this relationship [23, 29, 30]. Furthermore, smaller, rather than larger, plantar flexor muscles may be more favorable for long-distance running among endurance runners [31, 32]. Therefore, it is hypothesized that the relationship between plantar flexor muscle size and dorsiflexion flexibility is unique for sprinters and/or endurance runners. To aid in the application of the present findings to the clinical setting, further studies are needed to determine the effect of plantar flexor muscle size on dorsiflexion flexibility in various populations.

The present study has several limitations. First, in this study, we instructed the subjects to refrain from performing stretching exercises for at least 2 hours before the measurements of dorsiflexion flexibility parameters, as in our previous studies [4, 5]. This decision was based on findings of previous studies showing that the level of dorsiflexion flexibility increased after acute plantar flexor stretching exercises and reversed to that before stretching exercises within 30 min [11, 19, 22]. Nevertheless, some previous studies strictly prohibited stretching exercises for more than 2 hours [17, 18]. Additionally, we instructed the subjects to avoid strenuous physical activity within 24 hours before the dorsiflexion flexibility measurements, as in previous studies [20, 21]. However, this interval might be insufficient to measure joint flexibility correctly because some previous studies strictly prohibited strenuous physical activity for more than 24 hours (i.e., > 48 hours) [17, 18]. Next, although we measured the maximal angle achieved during voluntary dorsiflexion as active dorsiflexion ROM, this measure may be affected by dorsiflexor strength. Furthermore, Kubo et al. [9] reported a positive correlation between plantar flexor maximal voluntary torque and passive dorsiflexion stiffness. Thus, plantar flexor and dorsiflexor strength might be related to the joint flexibility parameters measured in the present study. However, we did not measure plantar flexor or dorsiflexor strength. Further studies are needed to comprehensively determine the relationships of the muscle size and strength of the plantar flexors and dorsiflexors with dorsiflexion flexibility.

## Conclusions

Using US and MRI, this study determined that a larger plantar flexor MV is correlated with less dorsiflexion flexibility, as assessed by active ROM, passive ROM, and passive stiffness. The findings of this study suggest that the plantar flexor muscle MV is related to dorsiflexion flexibility in healthy young males. Therefore, the present findings may be helpful in understanding the determinant factors of joint flexibility and in assessing the effect of flexibility training/rehabilitation among various populations.

## Data Availability

Data will be provided the corresponding author upon request.

## Abbreviations

ACSA: Anatomical cross-sectional area
CSA: Cross-sectional area
MT: Muscle thickness
MRI: Magnetic resonance imaging
MV: Muscle volume
ROM: Range of motion
US: Ultrasonography
